# Three Different Types of Fat Grafting for Facial Systemic Sclerosis: A Case Series

**DOI:** 10.3390/jcm11185489

**Published:** 2022-09-19

**Authors:** Antonio Arena, Umberto Committeri, Fabio Maglitto, Giovanni Salzano, Giovanni Dell’Aversana Orabona, Luigi Angelo Vaira, Pasquale Piombino, Michela Apolito, Gianluca Renato De Fazio, Luigi Califano

**Affiliations:** 1 Maxillofacial Surgery Operative Unit, Department of Neurosciences, Reproductive and Odontostomatological Sciences, Federico II University of Naples, 80131 Naples, Italy; 2 Division of Surgical Oncology Maxillo-Facial Unit, Istituto Nazionale Tumori-IRCCS-Fondazione G. Pascale, Via Mariano Semmola, 80131 Naples, Italy; 3 Maxillofacial Surgery Operative Unit, University Hospital of Sassari, 07100 Sassari, Italy

**Keywords:** systemic sclerosis, fat graft, orofacial SSc, maxillo-facial surgery, lipofilling

## Abstract

Systemic sclerosis (SSc) is a heterogeneous, chronic connective tissue disease, characterized by skin fibrosis as well as vascular and visceral lesions. It can involve the lungs, heart, kidneys, gastrointestinal tract, and bones. The orofacial manifestations of SSc can cause functional, aesthetic, and social distress, resulting in significant psychological implications for the patients. In recent decades, fat grafting improved the aesthetic outcomes in terms of volume deficiency, contour asymmetry, and skin elasticity of the face thanks to the regenerative action of the stem cells contained within it. We describe five cases of a patient with SSc treated with fat grafting used to correct volume loss and facial elasticity of the lips and perioral region on the middle and lower third of the face. All the patients received regular postoperative checks at weeks 1 and 2. A multiple choice questionnaire was administered to assess the degree of tolerability of the procedure. The reliability of the questionnaire was evaluated by calculating the Cronbach alpha using the MedCalc Statistical Software version 20.113. The aim of our study is to describe three different types of fat grafting used to correct volume loss and restore facial elasticity of the lips and perioral region on the middle and lower third of the face.

## 1. Introduction

Systemic sclerosis (SSc) is a heterogeneous, chronic connective tissue disease, characterized by skin fibrosis and vascular and visceral lesions. It involves the lungs, heart, kidneys, gastrointestinal tract, and bones [1]. 

Two subtypes with cutaneous involvement have been described: limited cutaneous SSc and diffuse cutaneous SSc. In both of them, the face is frequently affected, with related aesthetic and functional disorders [2,3].

Due to the loss of elasticity and skin fibrosis, the face’s involvement in SSc often causes a “mask appearance” and microstomy, compromising some regular daily activities, such as eating, talking, drinking, and practicing regular oral hygiene.

The orofacial manifestations of SSc can cause functional, aesthetic, and social distress, resulting in significant psychological implications for the patients.

As reported in the literature, in recent decades, fat grafting improved the aesthetic outcomes in terms of volume deficiency, contour asymmetry, and skin elasticity of the face, thanks to the regenerative action of the stem cells contained within it.

The aim of our study is to describe three different types of fat grafting used to correct volume loss and facial elasticity of the lips and perioral region on the middle and lower third of the face.

## 2. Materials and Methods

Five consecutive patients underwent fat grafting for SSc with aspects of diffuse cutaneous sclerosis between January 2021 and June 2021 at the Maxillo-Facial Surgery Unit of the University of Naples “Federico II”. All the patients were informed about the procedure and signed a preoperative consent for data recording in our clinical database. All patients underwent clinical facial analysis before the procedure. The patients presented the typical mask-like appearance of the face, with hypotrophy of the middle and lower third, labial incompetence with microstomy, and limited mouth opening. Hypoelasticity and thickening of the skin was highlighted, especially in the perioral and labial region. All of them mostly complained about difficulties in daily activities, such as talking, drinking, or eating, with frequent drooling, fatigue, and considerable psychological distress.

For these reasons, we decided to improve skin elasticity and volume loss through three different type of fat grafting. This study was approved by the Federico II University Ethics Committee (protocol N.81/20) on 2 April 2020.

### 2.1. Surgical Technique

The procedure was performed under general anesthesia, and the abdomen was the donor site. A modified Klein solution composed of 1 Lt saline solution with 100 mL of 1% plain lidocaine and 1 ml of 1:1000 adrenaline was injected into the donor site through a 14-gauge spiral, multi-port tumescent infiltrator cannula (Tulip Medical, San Diego, CA, USA) to obtain hydro-dissection and fat tumescence. An atraumatic liposuction using a 14 G × 15 cm Carraway Harvester with a spiral three-port design (Tulip Medical, San Diego, CA, USA) on a 20 mL luer-lock syringe with a low suction pressure allowed us to collect 90 cc of raw adipose tissue. The fat obtained was washed several times with a saline solution, and a gravity separation was achieved.

**Figure 1:**
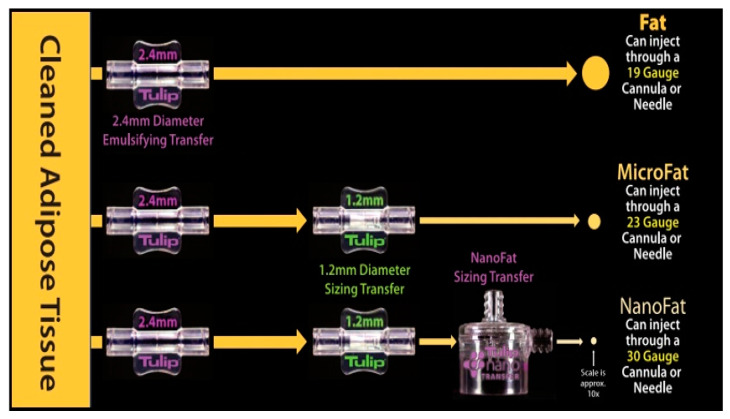
Harvested fat processing procedure sequence.

Three different modalities to process the harvested fat were performed (Figure 1):

Macrofat: 20 cc of fat obtained was transferred in a 3 cc luer-lock syringe through a Luer to Luer 2.4 mm Anaerobic Transfer (Tulip Medical, San Diego, CA, USA). 

Microfat: 15 cc of fat obtained was processed by an intersyringe shuffle using a Luer to Luer 2.4 mm Anaerobic Transfer (Tulip Medical, San Diego, CA, USA). After 20 passes, the same process was repeated through Luer-Lock 1.2 mm Connector (Tulip Medical, San Diego, CA, USA) for the other 15 passes.

Nanofat: the same two steps applied to obtain the microfat were performed starting from 20 cc of harvested fat. Using a 400-micron filter NanoTransfer (Tulip Medical, San Diego, CA, USA), the connective tissue remnants were removed. 

**Figure 2:**
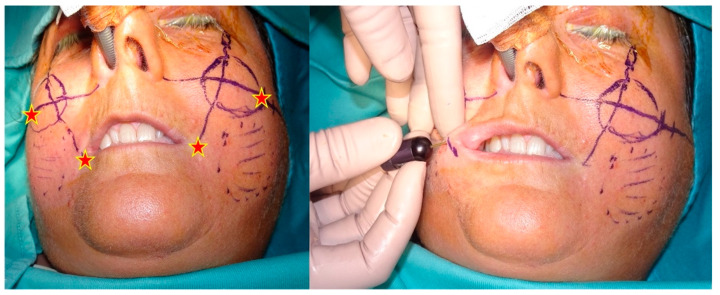
Surgical marking and entry points (indicated as pentagrams) of procedure.

**Figure 3:**
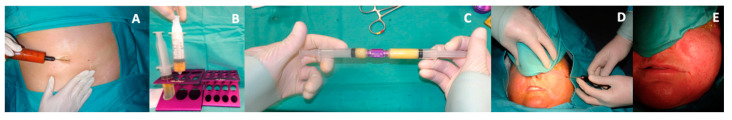
Fat collection from the abdominal region (**A**), fat decanting (**B**), fat processing (**C**), fat grafting (**D**), and the immediate postoperative state (**E**).

Macrofat was used to restore the volume of the lip, perioral rim, and zygomatic area after a skin and dermis penetration with an 18-gauge needle in four entry points. The entry points were symmetrical for both facial sides and were positioned 1 cm laterally in the labial commissure and in the area between the midcheek and the zygomatic arch (Figure 2 and Figure 3). 

Approximately 3.5 to 5 mL of fat per side was injected with a single orifice 18-gauge × 7 cm microinjector cannula (Tulip Medical, San Diego, CA, USA). The quantity of fat injected was based on the morphology of the patient’s face. Microfat was placed to improve the nasolabial folds and the cheekbone area through the same entry points used for the macrofat. An amount of 1.5 to 2.5 mL of fat per side was injected with a retrograde fan technique using a 20-gauge × 5 cm single orifice blunt cannula (Tulip Medical, San Diego, CA, USA). Nanofat was injected superficially in the perioral wrinkles and in the vermilion border using a 27-gauge needle. An amount of 2 to 3 mL of nanofat per side was injected using a retrograde linear technique.

### 2.2. Postprocedure Assessment

**Figure 4:**
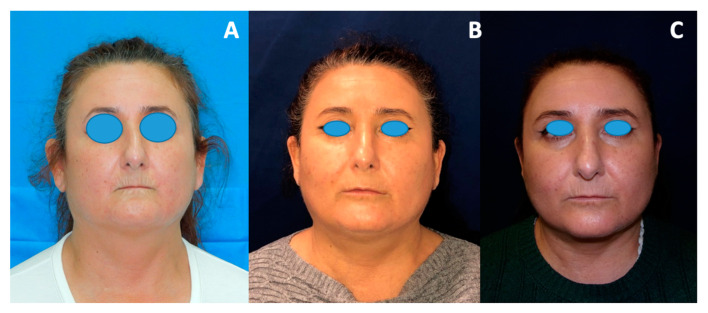
Preoperative (**A**), 6 months postoperative (**B**), 1 year postoperative (**C**).

All the patients received regular postoperative specific checks at 1 and 2 weeks as well as at 6 months and 1 year after the procedure (Figure 4). A multiple choice questionnaire was administered to assess the degree of tolerability of the procedure and the aesthetic and functional outcomes. A postoperative evaluation of pain and itching was requested 2 weeks after surgery; aesthetic and functional improvements were evaluated at the 6-month check.

The questionnaire was built and evaluated according to a 5-point Likert scale. The five items included the following:

1. Pain;
2. Itching;
3. Tissue elasticity;
4. Degree of aesthetic satisfaction;
5. Lip competence.

For each question, the patient could give a score from 1 up to 5 corresponding to the degree of tolerability. A score ranging from 20 to 25 was considered very good, from 15 to 19 was considered good, from 10 to 14 was considered acceptable, and <9 was considered poor. The reliability of the questionnaire was evaluated by calculating the Cronbach alpha using the software MedCalc Statistical Software version 20.113 (MedCalc Software bv, Ostend, Belgium; https://www.medcalc.org; accessed on 16 October 2020).

## 3. Results

In our sample, there were 5 patients (3F, 2M). No surgical complications were detected during outpatients’ checks. No other complications, such as allergic reactions or hematoma directly related with the procedure, were reported. 

**Figure 5:**
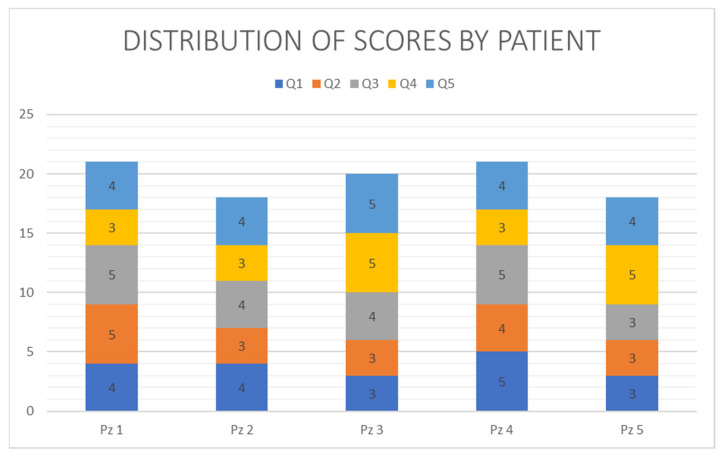
Distribution of score.

The reliability of the administered questionnaire was confirmed by the Cronbach alpha being equal to 0.83 with raw variables and 0.85 with standardized variables. The effect of the mask on the 5 items defined in the administered questionnaire is reported in Figure 5. The overall results are reported in Table 1. Three patients reported a very good result, giving a score between 20 and 25 (mean 20.67), and two patients reported a good result, giving a score between 15 and 19 (mean 18). The total average score was 19.6.

## 4. Discussion

SSc is a chronic autoimmune disease of unknown etiology, characterized by diffuse fibrosis, abnormal immune system activation, and skin, joint, and internal organ microvascular anomalies. In particular, the complications of the internal organs are the main causes of the high mortality rate. It is more common among women, especially between the ages of 20 and 50.

This disease is highly disabling for the patient due to the aesthetic and functional outcomes that limit daily life. The most frequent symptoms include Raynaud’s phenomenon, polyarthralgia, dysphagia, heartburn, swelling, and, eventually, retraction of the fingers and skin fibrosis. [1] Skin and visceral organ fibrosis is caused by the synthesis and deposition of the extracellular matrix and collagen determined by profibrotic myofibroblasts [4,5,6].

SSc can be divided into limited systemic sclerosis, formerly referred to as CREST syndrome (calcinosis of the skin, Raynaud’s phenomenon, esophageal motility disorders, sclerodactyly, telangiectasia); generalized systemic sclerosis (with widespread skin involvement); systemic sclerosis without scleroderma; and overlap syndromes that present the typical symptoms of other connectivitis.

The most evident feature of the full-blown disease is the presence of skin fibrosis, as indicated by the name scleroderma.

In the initial forms, it occurs only with edema. In advanced forms, the disease is characterized by loss of skin elasticity, smoothing of expression lines on the face, difficulty opening the oral rim, lip resorption (microcheilia), and difficulty completely moving the joints, especially the hands that tend to assume a curved (“claw”) attitude with hindrances in the extension and fine movements of the fingers.

Recently, the use of autologous fat grafting in SSc has allowed a reduction of the effects of skin fibrosis caused by the disease. As reported in the literature, fat grafting has already been used in various conditions such as scars, post-traumatic deformities, radiodermatitis, congenital anomalies, contour abnormalities, burn injuries, breast capsular contracture and augmentation, cosmetic procedures, and also localized forms of scleroderma such as “en coup de saber” [7,8,9,10]. As described for the first time by Coleman, we used the lipofilling technique on our patient, which consisted of taking a fat graft from a donor site (abdomen, thigh, buttock) to a recipient one [11]. Based on the technique of extraction and manipulation of the harvested tissue, we obtained three different types by size: macrofat graft for the subcutaneous tissue, microfat graft for the deep dermis, and nanofat graft for the superficial dermis. 

In particular, the macrofat was infiltrated for its volumizing effect. Microfat has been exploited for its regenerative and filling action in the cheekbone, lip, and nasolabial fold region [12]. The biorevitalizing effect of nanofat improved perioral wrinkles and the motility of the lips, increasing the mouth opening. During the procedure, cannulas and needles of different sizes were used in the different facial areas due to the type of injection.

In addition to its filling effect, the high number of mesenchymal stem cells (ASCs) obtained with this technique represent the major advantage compared to any other body tissue graft [13]. The regenerative power of ASCs is attributable to their ability to secrete angiogenic factors and immunomodulatory properties that facilitate tissue repair [14,15].

The limit of the procedure is represented by the survival time of the grafted fat and by the long follow-up, both due in part to the reabsorption of the adipose tissue and to the long times of differentiation of the stem cells and of the angiogenic processes [16].

## 5. Conclusions

In conclusion, this surgical technique resulted in a greater elasticity of the skin tissue, a smoothing of the perioral wrinkles, and an improvement in microstomy, drooling, and fatigue. On the other hand, no effect was found on the increase in the volume of the zygomatic region and the lips, probably due to the reduced follow-up and partial reabsorption of the tissue. 

To achieve the ideal volume and more evident results, which can improve the quality of life in carrying out daily activities, repeated injections are indicated [17,18].

## Figures and Tables

**Table 1:** Patients’ questionnaire answers.

	Q1	Q2	Q3	Q4	Q5
Pz 1	4	5	5	3	4
Pz 2	4	3	4	3	4
Pz 3	3	3	4	5	5
Pz 4	5	4	5	3	4
Pz 5	3	3	3	5	4

## Data Availability

The data presented in this study are available on request from the corresponding author. The data are not publicly available due to privacy.
